# Evaluation of effect of body mass index and weight loss on survival of patients with nasopharyngeal carcinoma treated with intensity-modulated radiation therapy

**DOI:** 10.1186/s13014-015-0443-3

**Published:** 2015-06-30

**Authors:** Yu-Hsuan Lin, Kuo-Ping Chang, Yaoh-Shiang Lin, Ting-Shou Chang

**Affiliations:** Department of Otolaryngology, Head and Neck surgery, Kaohsiung Veterans General Hospital, No.386, Ta-Chung 1st Rd., Kaohsiung, 813 Taiwan; Department of Otolaryngology, Head and Neck surgery, National Defense Medical Center, Taipei, Taiwan; Institute of Public Health, College of Medicine, National Cheng Kung University, Tainan, Taiwan

**Keywords:** Nasopharyngeal carcinoma, Intensity-modulated radiation therapy, Body mass index, Weight loss, Malnutrition

## Abstract

**Background:**

Previous studies report body-mass index (BMI) and percent weight loss (WL) to have prognostic significance when treating patients with nasopharyngeal carcinoma (NPC). However, most of these investigations studied patients treated using different radiotherapeutic techniques. We evaluated the predictive effect of these two nutrition-related measurements on therapeutic outcome in NPC patients who only received intensity-modulated radiation therapy (IMRT) as part of their total treatment program.

**Methods:**

We retrospectively studied NPC patients treated with IMRT from January 2006 to February 2012. Cox proportional hazards was used to test the association of pretreatment BMI (<23 kg/m^2^ vs. ≥23 kg/m^2^) and percent weight loss (≥5 % vs. <5 %) during therapy and related survival rates while controlling for various potential confounders.

**Results:**

Eighty-one (34 %) of the 238 patients had BMIs ≥23 kg/m^2^ at pretreatment and 150 (63 %) had significant (≥5 %) weight loss. Median follow-up time was 41.71 months; median radiotherapy was 7.46 ± 0.77 weeks. Those with BMIs ≥23 kg/m^2^ did not have a better 3-year overall survival (*p* = 0.672), 3-year disease specific survival (*p* = 0.341), 3-year locoregional free survival (*p* = 0.281), or 3-year distant metastatic free survival (*p* = 0.134). Those with significant WL (≥5 %) did not have worse 3-year clinical endpoints, even after stratifying magnitude of weight loss by BMI category. In sensitivity test, the adjusted hazard ratio remained statistically insignificant using different cutoffs for BMIs and percent weight loss.

**Conclusions:**

This study found no significant relationship between BMI and percent weight loss on survival of NPC patients receiving IMRT based therapy. Further studies might want to consider other nutrition related factors as prognostic indicators when studying the correlate between malnutrition and survival in this population.

**Electronic supplementary material:**

The online version of this article (doi:10.1186/s13014-015-0443-3) contains supplementary material, which is available to authorized users.

## Introduction

Nasopharyngeal carcinoma(NPC) is a head and neck epithelial malignancy with a striking racial/ethnic distribution and endemic to Southeast Asia and southern China. It differs from non-nasophayngeal head and neck squamous cell carcinoma in several ways, including its etiological association with Epstein-Barr virus, high radio- and chemo-sensitivity, and a greater propensity for presenting as locoregional advanced disease at diagnosis [1, 2]. Its treatment has been enhanced greatly by intensity-modulated radiotherapy (IMRT), which has improved locoregional control but not distant metastasis [3]. The IMRT technique might be improved by the identification of predictors of poorer prognosis among these patients. Although several promising molecular targets have been found to predict treatment failure in NPC patients, these tests take too much time to perform and are not routinely tested by most medical institutes.

Malnutrition has been significantly and directly associated with overall survival among cancer patients [4]. Body mass index (BMI) and weight change during therapy, two nutrition-related factors, have been found to have prognostic significance in NPC. In particular, several studies have reported low pretreatment BMI and a high weight loss during therapy to be independently and significantly associated with poorer survival independent of several established factors [5–7]. However, the subjects of these studies were patients treated with diverse radiotherapeutic techniques combined together in one study group. The techniques used to treat NPC change over time and vary regionally, and thus it may be necessary to reevaluate the prognostic values of certain factors when one technique replaces others as a preferred means of treatment. In this study, we wanted to find out if two previously reported anthropometric measurements, pretreatment BMI and weight loss, remained predictive of prognosis in NPC patients being treated in a program using IMRT based therapy. To do this, we retrospectively recruited 238 consecutive patients with nasopharyngeal carcinoma treated with IMRT in a single medical institute, and studied the effect of pretreatment BMI and weight loss during therapy on overall survival, disease-specific survival, locoregional free survival, and distant metastasis free survival, controlling for various related factors.

## Materials and methods

### Patients and data collection

The protocol for this study was approved by the Institutional Review Board of Kaohsiung Veterans General Hospital, Taiwan. We enrolled 260 consecutive patients newly diagnosed with nasopharyngeal carcinoma at Kaohsiung Veterans General Hospital from January 2006 to February 2012. All patients had received routine magnetic resonance imaging for tumor staging. Patients were excluded if they were below 18 years old (*n* = 1), were classified as having a World Health Organization (WHO) classification type one lesion (*n* = 5), or were found to have distant metastasis at initial diagnosis or had any other malignancy treated with radiotherapy previously or concomitantly (*n* = 16). After exclusion, we were left with a total of 238 patients to include in our analysis.

### Nutritional data

Pretreatment weight was measured on day one of radiotherapy for patients receiving radiotherapy alone or concurrent radiotherapy or on day one chemotherapy for patients receiving induction chemotherapy prior to radiotherapy. The post-treatment weight was measured one month following treatment. A critical weight loss was defined as weight loss greater than 5 %, as of this magnitude is considered to indicate possible nutritional deterioration [8, 9]. Baseline BMI prior to radiotherapy was calculated by dividing the pretreatment weight (kgs) by the square of height (meters). The patients were further divided into two groups using the cut-off point, 23 kg/m^2^, a WHO classification for overweightness and obesity for Asians [10].

### Radiotherapy

Before treatment, all patients were immobilized with a thermoplastic head and shoulder mask, and CT simulation was performed following standard procedures. All patients were treated with IMRT technique. The total prescribed dose was 70–76 Gy to the gross tumor volume of nasopharynx (GTVnx) and the gross tumor volume of positive neck lymph nodes (GTVnd), 60–66 Gy to the high risk region as clinical target volume 1 (CTV 1), and 50–60 Gy to low risk region as CTV2. The radiation given to both gross tumor and regional lymphatics was administrated in a conventional fractionated dose of 1.6–2 Gy, one fraction per day, five days per week.

### Chemotherapy

Our institution recommends radiotherapy only for patients in stages I–II, and concurrent chemoradiotherapy for those in stages III–IVB. Induction chemotherapy was administered to patients with T4 and/or N3 disease, whereas adjuvant chemotherapy was administered to patients with residual disease of high incidence for distant metastasis, including N3, T3/T4 with multiple neck lymph nodes metastasis, multiple neck lymphadenopathy with one of node size > 4 cm [11]. Induction and adjuvant chemotherapy bolus injections of 80 mg/m^2^ of cisplatin on day one were followed by 1000 mg/m^2^ of fluorouracil administered daily by 96-h continuous infusion from Day 2 to Day 5 every 3 to 4 weeks. The concurrent chemotherapy was prescribed for 188 (79 %) patients at a dosage of 80 to 100 mg/m^2^ of cisplatin on Day one, Day 22, and Day 43 or 30 mg/m^2^ of cisplatin every week, for 6–8 cycles. After completion of treatment, further follow-up assessments were performed at 3-month intervals for the first three years. The intervals gradually increased to every four months, biannually to annually, thereafter.

### Clinical end points

Clinical endpoints were 3-year overall survival, disease specific survival, any recurrence or distant metastasis. Overall survival was defined as the time that had elapsed between the diagnosis and the date of death from any cause or three years if patient was still alive at the end of the study period. Disease specific survival, locoregional free survival, and distant metastasis free survival were otherwise calculated from the start of radiotherapy. Failure free survival (FFS) was defined as the time that had elapsed between the initiation of radiotherapy and the date of recurrence and/or metastasis or three years if patient was free of disease. Patients who were lost to follow-up within 3 years were censored at their last date of follow-up.

### Statistical analysis

All statistical operations were performing using the SPSS ver. 15 (SPSS Inc., Chicago, IL, USA). Pearson’s chi-square tests were used to explore the differences between categorical variables and *t*-test for continuous variables. Overall survival, disease specific survival, distant metastasis free survival, and locoregional free survival were generated according to the methods of Kaplan and Meier. Differences between survival curves were compared using the log-rank test. The prognostic influence of pretreatment BMI and weight loss during therapy were assessed using Cox proportional hazards multivariate model after adjusting for patients’ characteristics, including age, gender, AJCC T classification, AJCC N classification, category of Charlson Comorbidity Index Score, smoking status, education level (low being less than junior high school, medium being high school level, and high being one year or more of college), treatment modalities (RT alone, CCRT, RT/CCRT + CT), and hemoglobin level. A two-sided *p*-value < 0.05 was considered significant. To explore whether the results would hold against changes in the cut-off values, we conducted a sensitivity analysis utilizing three other combinations of widely used cutoffs for BMI (kg/m^2^) and critical weight loss (%) [6, 8, 12]: (1) 23 kg/m^2^ and 10 % (2) 25 kg/m^2^ and 5 %, and (3) 25 kg/m^2^ and 10 %.

## Results

### Demographic data and clinical characteristics

Patient characteristics are described in Table 1. In total, there were 238 patients predominately male (71 %). The median age was 50.26 ± 11.82 years. Two hundred and two patients (84.9 %) were categorized as having stages III and IVA/IVB disease based on the 7^th^ UICC/AJCC staging system. Mean BMI was 24.75 ± 4.19. Eighty-one patients (34 %) had BMIs below 23 kg/m^2^, and 157 (66 %) had BMIs greater than 23 kg/m^2^. The average weight loss from first radiotherapy to one month post-treatment was 7.85 ± 4.32 kgs. Eighty-eight patients (37 %) had weight loss <5 % and 150 (63 %) had weight loss ≥5 %. A greater proportion of the patients with pretreatment BMI ≥ 23 kg/m^2^ were more likely to have high weight loss. Subjects with pretreatment BMIs ≥23 kg/m^2^ had higher serum hemoglobin (*p* = 0.001). However, no significant differences were found in either category of pretreatment BMI regardless of CCIS group, treatment arm, or education level. Forty-one patients (17.2 %) had radiotherapy only, and the rest received combined modality treatment. The median duration of radiotherapy was 7.46 ± 0.77 weeks and the median follow-up time was 41.71 months.

**Table 1:** Clinical data of 238 nasopharyngeal carcinoma patients

		Pre-treatment BMI, kg/m^2^		
Variable	All	<23 (*N* = 81)	≥23 (*N* = 157)	*p*
Age, y	50.26 ± 11.82	49.02 ± 12.84	50.09 ± 11.24	0.246
Sex (%)				<0.001
male	169(71.0)	45(55.6)	124(79.0)	
female	69(29.0)	36(44.4)	33(21.0)	
Smoke (%)				0.106
No	160(67.2)	60(74.1)	100(63.7)	
Yes	78(32.8)	21(25.9)	57(36.3)	
CCIS (%)				0.742
0	184(77.3)	64(79.0)	120(76.4)	
1	32(13.4)	9(11.1)	23(14.6)	
≥2	22(9.2)	8(9.9)	14(8.9)	
Histology type				0.082
NUC	214(89.9)	69(85.2)	145(92.4)	
NDC	24(10.1)	12(14.8)	12(7.6)	
T classification (%)				0.253
T1/T2	121(50.8)	37(45.7)	84(53.5)	
T3/T4	117(49.2)	44(54.3)	73(46.5)	
N classification (%)				0.779
N0/N1	62(26.1)	22(27.2)	40(25.5)	
N2/N3	176(73.9)	59(72.8)	117(74.5)	
AJCC Stage (%)				0.775
Stage 1/2	36(15.1)	13(16.0)	23(14.6)	
Stage 3/4	202(84.9)	68(84.0)	134(85.4)	
Treatment arm (%)				0.592
RT alone	41(17.2)	15(18.5)	26(16.6)	
CCRT alone	93(39.1)	28(34.6)	65(41.4)	
RT/CCRT + CT	104(43.7)	38(46.9)	66(42.0)	
BWL (%)				0.003
<5 %	88(37.0)	41(50.6)	47(29.9)	
≥5 %	150(63.0)	40(49.4)	110(70.1)	
Education level (%)				0.97
Low	113(47.5)	39(48.1)	74(47.1)	
Medium	70(29.4)	23(28.4)	47(29.9)	
High	55(23.1)	19(23.5)	36(22.9)	
RT duration	7.46 ± 0.77	7.58 ± 0.80	7.40 ± 0.75	0.091
Hemoglobin	13.85 ± 1.77	13.28 ± 1.66	14.15 ± 1.75	0.001

CCIS Charlson Comorbidity Index Score; NUC nonkeratinizing undifferentiated carcinoma; NDC nonkeratinizing differentiated carcinoma; BMI body mass index; BWL body weight loss; RT radiotherapy; CT chemotherapy; CCRT concurrent chemoradiotherapy

Values are numbers (percentage)

### Univariate survival analysis

**Fig. 1:**
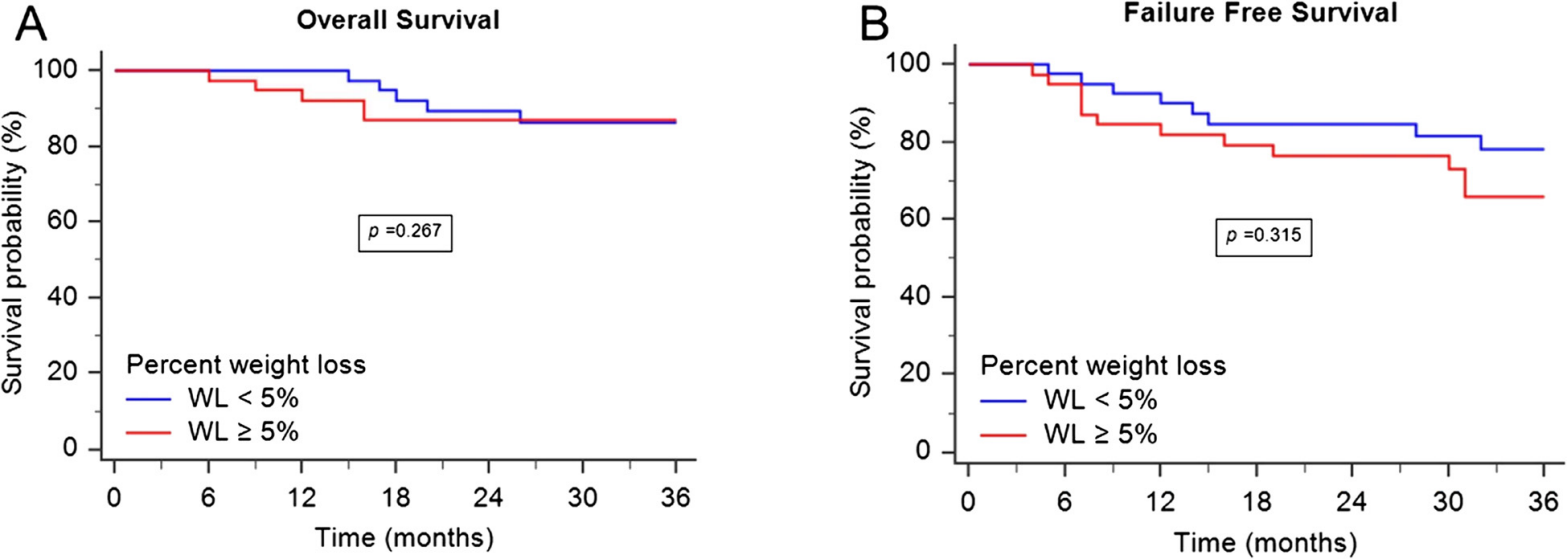
OS and FFS for 157 NPC patients with BMI ≥ 23. **a** Patients with weight loss greater than 5 % did not have a worse 3-year overall survival (*p* = 0.267). **b** There was no difference with respect to the 3-year failure free survival between the two groups (*p* = 0.315)

**Figure 2:**
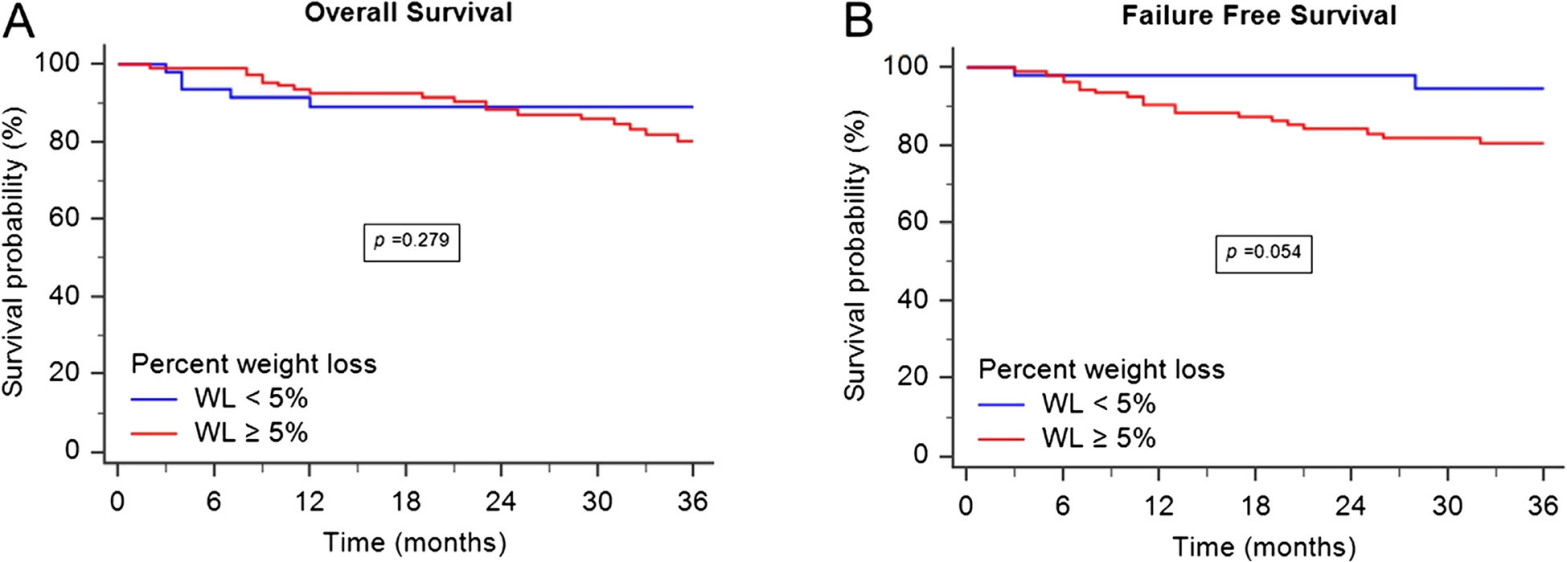
OS and FFS for 81 NPC patients with BMI < 23. **a** Patients with weight loss percentage greater than 5 % demonstrated no superior 3-year overall survival (*p* = 0.279). **b** The difference of 3-year failure-free survival between the two groups was not statistically significant (*p* = 0.054)

As can be seen in Table 2, neither pretreatment BMI nor percent weight loss was significantly associated with overall survival, disease specific survival, distant metastasis free survival, or locoregional free survival. In the 157 NPC patients with BMIs ≥ 23, those with weight loss < 5 % had no survival benefit in overall survival and failure-free survival (Fig. 1). Similarly, percent weight loss had no impact on the survival of 81 NPC patients with BMIs <23 (Fig. 2). Comorbidity was associated with lower three-year overall survival (*p* = 0.025) and locoregional free survival (*p* = 0.022). In addition, patients with advanced AJCC N classification disease had poorer three-year disease specific survival (*p* = 0.024).

**Table 2:** Univariate analyses of risk factors for 3-year LRFS, DMFS, DSS, and OS rates

Variable	LRFS (%)	*p*	DMFS (%)	*p*	DSS (%)	*p*	OS (%)	*p*
Age (y)		0.165		0.911		0.198		0.042
<50	91.9		88.0		92.0		88.8	
≥50	88.6		87.3		84.9		87.0	
Sex		0.980		0.032		0.078		0.880
Male	90.8		91.3		90.4		84.5	
Female	88.6		79.4		83.5		81.8	
preT BMI		0.466		0.052		0.213		0.667
<23 kg/m^2^	86.9		81.1		86.5		86.7	
≥23 kg/m^2^	91.9		93.8		89.2		82.0	
Education level		0.580		0.530		0.130		0.092
Low	92.6		86.1		84.5		78.2	
Medium	88.1		87.1		86.7		82.8	
High	87.4		91.8		98.0		96.2	
Smoke		0.463		0.257		0.318		0.828
No	90.9		86.4		86.6		83.1	
Yes	88.4		90.5		91.9		84.9	
CCIS		0.022		0.638		0.663		0.025
0	93.9		86.7		89.5		85.4	
1	69.9		92.9		84.7		82.0	
≥2	88.8		89.3		83.8		71.9	
T stage		0.317		0.928		0.474		0.194
T1/T2	92.2		86.9		89.8		85.7	
T3/T4	87.9		88.7		86.5		81.5	
N stage		0.657		0.138		0.024		0.134
N0/N1	92.4		92.8		96.6		89.7	
N2/N3	89.3		85.8		85.3		81.4	
Treatment		0.488		0.013		0.094		0.471
RT alone	93.8		86.9		92.3.		89.9	
CCRT alone	91.8		94.9		93.0		83.5	
RT/CCRT + CT	85.4		84.2		81.7		80.6	
BWL during RT		0.111		0.097		0.256		0.113
≥5 %	100.0		92.7		90.2		86.1	
<5 %	88.2		84.7		90.7		82.1	
Hemoglobin		0.652		0.225		0.364		0.206
≥13.5	91.5		90.1		89.0		85.7	
<13.5	88.3		83.0		87.4		79.2	

*Abbreviations*: LRFS locoregional-free survival; DMFS distant metastasis-free survival; DSS disease-specific survival; OS overall survival. Other abbreviations as in Table 1

### Multivariate survival analysis

Multivariate analysis also showed that pretreatment BMI and percentage of weight loss were not associated with all-cause or cause-specific mortality (Table 3 and Table 4). Still after adjustment for cofounders, multivariate analysis revealed a significant difference in three-year overall survival between patients with CCISs ≥2 and those without comorbidity (hazard ratio [HR], 3.043 [95 % confidence interval {CI}, 1.27–7.31; *p* = 0.013]) and between patients with high education levels and those low education level (0.360, [95 % CI, 0.13–0.99; *p* = 0.047]). In addition, compared to patients without comorbidities, those with CCIS = 1 tended to have locoregional recurrence (4.264, [95 % CI, 1.56–11.65; *p* = 0.005]). The results remained statistically insignificant after a sensitivity analysis using different cutoffs (Additional file 1: Appendix S1). In summary, significant weight loss did not confer a worse 3-year survival rate after stratifying for magnitude of weight loss by BMI category.

**Table 3:** Multivariate analysis for OS and DSS for all patients

		OS		DSS	
Variable	Comparison	*p*	HR (95 % CI)	*p*	HR (95 % CI)
Age	Young vs. old	0.141	1.715(0.84–3.52)	0.204	1.756(0.74–4.19)
Sex	Male vs. female	0.786	1.100(0.55–2.20)	0.194	1.727(0.76–3.94)
preT BMI	<23 kg/m^2^ vs. ≥23 kg/m^2^	0.672	1.164(0.58–2.36)	0.341	0.670(0.29–1.53)
Education level	Low vs. Medium	0.427	0.731(0.34–1.58)	0.807	1.122(0.44–2.84)
	Low vs. high	0.047	0.360(0.13–0.99)	0.199	0.368(0.08–1.69)
CCIS	0 vs. 1	0.852	0.911(0.34–2.42)	0.660	1.282(0.42–3.88)
	0 vs. ≥2	0.013	3.043(1.27–7.31)	0.318	1.936(0.53–7.08)
T classification	Early vs. late	0.191	1.563(0.80–3.06)	0.706	1.171(0.52–2.66)
N classification	Early vs. late	0.331	1.539(0.65–3.67)	0.128	3.206(0.71–14.40)
Treatment	RT vs. CCRT	0.595	1.325(0.47–3.74)	0.468	0.612(0.16–2.30)
	RT vs. RT/CCRT + CT	0.655	1.276(0.44–3.73)	0.840	1.132(0.34–3.79)
BWL percentage	<5 % vs. ≥5 %	0.087	1.910(0.91–4.01)	0.232	1.746(0.70–4.36)

*Abbreviations*: HR hazard ratio; CI confidence interval. Other abbreviations as in Tables 1 and 2

**Table 4:** Multivariate analysis for LRFS and DMFS for all patients

		LRFS		DMFS	
Variable	Comparison	*p*	HR (95 % CI)	*p*	HR (95 % CI)
Age	Young vs. old	0.085	2.395(0.89–6.47)	0.661	1.203(0.53–2.74)
Sex	Male vs. female	0.531	1.379(0.50–3.77)	0.145	1.864(0.81–4.30)
preT BMI	<23 kg/m^2^ vs. ≥23 kg/m^2^	0.281	0.601(0.24–1.52)	0.134	0.528(0.23–1.22)
Education level	Low vs. Medium	0.161	2.197(0.73–6.61)	0.538	1.336(0.53–3.36)
	Low vs. high	0.190	2.208(0.68–7.22)	0.750	0.823(0.25–2.72)
CCIS	0 vs. 1	0.005	4.264(1.56–11.65)	0.509	0.610(0.14–2.65)
	0 vs. ≥2	0.585	1.562(0.31–7.76)	0.804	1.217(0.26–5.73)
T classification	Early vs. late	0.444	1.437(0.57–3.63)	0.520	0.765(0.34–1.73)
N classification	Early vs. late	0.998	0.998(0.32–3.09)	0.524	1.448(0.46–4.53)
Treatment	RT vs. CCRT	0.592	1.549(0.31–7.68)	0.091	0.310(0.08–1.21)
	RT vs. RT/CCRT + CT	0.353	2.202(0.42–11.65)	0.893	1.081(0.35–3.34)
BWL percentage	<5 % vs. ≥5 %	0.284	1.759(0.63–4.94)	0.097	2.280(0.86–6.05)

*Abbreviations*: HR hazard ratio; CI confidence interval. Other abbreviations as in Tables 1 and 2

## Discussion

This study did not find an association between pretreatment BMI or magnitude of weight loss and clinical outcome in NPC patients receiving IMRT-based therapy. To the best of our knowledge, this study represents first study to investigate the effect of these parameters on in NPC patients receiving IMRT as the only radiotherapy in their treatment programs.

Previous studies have found both weight loss and BMI to be predictive of survival. Van Bokhorst-de van der Schuer et al. [13] reported that in patients with head and neck cancer (HNC) there was a direct association between weight at diagnosis and clinical outcome. Similarly, Capuano et al. [14] and Langius et al. [9] have reported that weight loss during (chemo)radiotherapy is a major predictor in this patient population. With regard to BMI, Bhaskaran et al. [15] in a robust study of the relation between BMI and a larger number of different cancers but not NPC, found that it had various levels of impact on the incidence and survival of several site-specific cancers. Park et al. [16] later observed a significant association between a higher BMI and longer survival in Korean patients with HNC and esophageal cancer. Pai et al. [17] and Ottosson et al. [12] reported similar findings for Chinese and Swedish patients with HNC.

NPC differs from HNC with regard to etiology, geographic distribution, racial distribution, and patient characteristics. In retrospective studies, Qiu et al. [18] and Ng et al. [19] reported a high prevalence of severe weight loss in NPC patients during radiotherapy, and Shen et al. [5] reported that such weight loss had a negative impact on prognosis in this population regardless of BMI category. One prospective study of NPC patients by Huang et al.[6] and a retrospective study Shen et al. [7] focusing on the pretreatment lifestyle behaviors and clinical outcome in NPC patients both found significant associations between BMI category and prognosis of locoregional advanced NPC. However, one problem with these investigations is that the radiation techniques used within each of their studies differed [5–7, 18, 19].

Our study did not find an association between percent weight loss or pretreatment BMI with prognosis in NPC. Our study was different from those mentioned above in that we studied NPC patients receiving IMRT based therapy and controlled for many possible confounding factors, so there may be several reasons for the difference in our findings and theirs. The first is that IMRT, unlike conventional 2D and conformational 3D radiation techniques, makes it more likely that the patient will complete his or her treatment plan within the planned time without interruption. IMRT can do this because it delivers a highly conformal dose to an irregular-shaped tumor making it possible to lower integral dose to organs at risk and normal critical tissues [20]. This reduced toxicity is responsible for IMRT’s improvements in radiotherapy-related xerostomia as well as its reduction of many of the most dramatic acute side effects of radiotherapy, including mucositis, pharyngitis, cutaneous desquamation of neck, and dysgusia, as it can be performed with tightly planned dose constraints [20, 21]. This reduced toxicity can alleviate some of the negative dietary symptoms that can lead to malnutrition and debilitation, when IMRT treatment is coupled with the aid of antiemetic agents, oral analgesics, and frequent oral rinsing [20, 21].

Furthermore, the reduced toxicity that comes with the use of IMRT may lessen the number and severity of head and neck symptoms and improve quality of life, according to Fang et al. [22, 23], who compared the effect of various radiotherapeutic tecnhiques on quality of life. Such improvements are believed to contribute to fewer treatment interruptions and better therapeutic tolerance. In the present study, all the patients completed the therapy within amount of time planned without interruption. It is possible that the uninterrupted completion of the treatment program and the reduction of adverse nutritional effects made possible by IMRT translated into better clinical outcomes for our patients, even in those with greater weight loss and lower pretreatment BMIs [24, 25].

Apart from the technical superiority of IMRT, the second reason our findings are different from the previous studies could be related to the possibility that pretreatment BMI or weight loss may not be adequate indicators of malnutrition in this patient group being treated with IMRT. Most of the HNC and NPC studies mentioned above have used BMI and weight loss as surrogates of malnutrition [5–7, 9, 12, 14, 17–19]. However, some studies have associated impairment of several other nutritional factors with poorer prognosis in NPC patients [26–29] and with quality of life in other populations [30, 31]. We assumed that because radiotherapy causes similar adverse responses in HNC and NPC patients, BMI and weight loss should have prognostic significance. However we did not find them to have this prognostic significance in our study.

BMI and weight loss may not be the best surrogates of malnutrition. Changes in body composition such as fat-free mass, especially muscle mass, and body fat mass, rather than pure total body weight may be more relevant to survival. For example, in their studies of patients treated for cancers of the respiratory and digastric tracts, Prado et al. [32] and Martin et al. [33] reported that low muscle mass (sacropenia) predicted poor survival and poor quality of life and survival independently of other covariates. In addition, muscle mass is known to vary within each BMI category, so cancer patients in any category of BMI may appear well or unwell regardless of percent weight loss [32–34]. As a consequence, considering the discrepancies between the two traditional anthropometric parameters and body composition in cancer patients, compartments of body composition might be more suitable indices of malnutrition for NPC population.

The prescription of nutritional supplementation, particular for cancer patients, has recently attracted the attention of the physicians treating them. Although there is consistent evidence that the provision of nutrients for cancer populations undergoing treatment predicts a less weight loss, fewer treatment-related complications, and a better quality of life [35], the impact of nutrition support on survival outcome remains controversial. Fearon et al. [36] reported a correlation between an effective nutrition intervention and better survival and attributed it to the maintenance of muscle mass. In contrast, Rabinovitch et al. [37] observed that patients with advanced-staged HNC receiving nutritional support prior to (chemo)radiotherapy had poorer locoregional control and overall survival. The current study lacked detailed information on the nutrients being used by the patients. Therefore, further research may be needed to determine the prognostic significance of nutritional intervention on NPC survival.

This study has some limitations. One limitation is that it is a hospital-based retrospective study with a small sample size. Another limitation is that we did not take into consideration the impact of chemotherapy on the nutritional measures and the resulting effect on prognosis. Another limitation was that BMI and weight loss measurement were limited to pre-treatment and treatment periods. Those measures can change after treatment and affect eventual health outcomes. Still another limitation is that we only used the traditionally studied BMI and percent weight loss as surrogates of malnutrition. We did not use other nutrition related factors to assess nutritional status, so it is not certain whether or not or to what extent our patients were indeed malnourished. In addition, it is worth noting that not all variables with significant *p*-value after a multiple testing truly indicate a significant difference. Due to the small number of samples and the unequal distribution of sample sizes, the significance of these variables should be interpreted with caution.

## Conclusions

This study found no significant relationship between BMI and percent weight loss on survival of NPC patients being treated with IMRT as part of their treatment program, possibly suggesting the use of IMRT reduces the importance of the traditional predictors of prognosis. Further studies may be needed to determine whether other nutrition related factors might better indicate nutritional state and correlate with survival in NPC patients.

## Additional file

Additional file 1: Appendix S1. Multivariate testing of sensitivity analysis using different cutoffs for BMIs and percent weight loss.

## Abbreviations

NPC: Nasopharyngeal carcinoma
NUC: Nonkeratinizing undifferentiated carcinoma
NDC: Nonkeratinizing differentiated carcinoma
HNC: Head and neck cancer
BMI: Body-mass index
preT BMI: Pretreatment body mass index
BWL: Body weight loss
IMRT: Intensity-modulated radiation therapy
AJCC: American Joint Cancer Council
CCIS: Charlson Comorbidity Index Scoring
WHO: World Health Organization
GTV: Gross tumor volume
CTV: Clinical target volume
OS: Overall survival
DSS: Disease specific survival
LRFS: Locoregional free survival
DMRS: Distant metastasis free survival
FFS: Failure free survival
RT: Radiotherapy
CCRT: Concomittent chemoradiotherapy
CT: Chemotherapy
HR: Hazard ratio
CI: Confidence interval
