# Supplementary material for: Evaluation of effect of body mass index and weight loss on survival of patients with nasopharyngeal carcinoma treated with intensity-modulated radiation therapy
Source: Radiat Oncol. 2015 Jun 30;10:136. doi: 10.1186/s13014-015-0443-3 (PMC4486696; doi:10.1186/s13014-015-0443-3)
Supplement: Additional file 1: Appendix S1. — Multivariate testing of sensitivity analysis using different cutoffs for BMIs and percent weight loss. [file 13014_2015_443_MOESM1_ESM.doc]

|  |  | **OS** | |  | **DSS** | |  | **LRFS** | |  | **DMFS** | |
| --- | --- | --- | --- | --- | --- | --- | --- | --- | --- | --- | --- | --- |
| **Variables** | **Comparison** | ***p*** | **Adjusted HR**  **(95% CI)** |  | ***p*** | **Adjusted HR**  **(95% CI)** |  | ***p*** | **Adjusted HR**  **(95% CI)** |  | ***p*** | **Adjusted HR**  **(95% CI)** |
| preT BMI | <23 vs. ≥23 | 0.576 | 1.219  (0.608-2.444) |  | 0.363 | 0.686  (0.304-1.546) |  | 0.385 | 0.670  (0.271-1.653) |  | 0.145 | 0.544  (0.240-1.234) |
| BWL percentage | <10% vs. ≥10% | 0.170 | 1.558  (0.827-2.932) |  | 0.219 | 1.652  (0.742-3.678) |  | 0.786 | 1.135  (0.456-2.825) |  | 0.068 | 2.107  (0.946-4.689) |
|  |  |  |  |  |  |  |  |  |  |  |  |  |
| preT BMI | <25 vs. ≥25 | 0.401 | 1.318  (0.691-2.513) |  | 0.631 | 0.817  (0.358-1.865) |  | 0.133 | 0.454  (0.162-1.273) |  | 0.731 | 0.868  (0.386-1.949) |
| BWL percentage | <5% vs. ≥5% | 0.118 | 1.819  (0.860-3.846) |  | 0.252 | 1.712  (0.682-4.297) |  | 0.225 | 1.919  (0.670-5.494) |  | 0.138 | 2.097  (0.788-5.580) |
|  |  |  |  |  |  |  |  |  |  |  |  |  |
| preT BMI | <25 vs. ≥25 | 0.308 | 1.394  (0.736-2.638) |  | 0.658 | 0.830  (0.365-1.890) |  | 0.198 | 0.514  (0.187-1.416) |  | 0.771 | 0.887  (0.397-1.984) |
| BWL percentage | <10% vs. ≥10% | 0.212 | 1.499  (0.794-2.830) |  | 0.233 | 1.633  (0.730-3.653) |  | 0.685 | 1.213  (0.478-3.079) |  | 0.094 | 1.982  (0.890-4.411) |

**Additional file 1: Appendix S1 Multivariate testing of sensitivity analysis using different cutoffs for BMIs and percent weight loss**

*Abbreviations*: Adjusted HR = adjusted hazard ratio; CI = confidence interval. Other abbreviations as in Tables 1 and 2.

*Adjusted for the patient’s age, sex, education level, Charlson Comorbidity Index Score, T classification, N classification, treatment modality
